# Second Report of the Hyderabad Chloroform Commission

**Published:** 1890-02-01

**Authors:** 


### SURGERY.

### SECOND REPORT OF THE HYDERABAD CHLOROFORM COMMISSION.

A notice of the first report of the Hyderabad Chloroform Commission appeared in the Hospital "Retrospect" for November 30th last. As the views of the commission and of Dr. Laurie (the principal medical officer of H.H. the Nizam's Government, under whose auspices the committee conducted experiments) were opposed to those generally received, the Nizam's Government gave the sum of £1,000 towards the expenses of a second investigation and a second report. The editors of the *Lancet* appear to have had the selection of a British member, and the choice fell upon Dr. Lauder Brunton, at which choice no one can cavil. But if Dr. Lauder Brunton, on going out to India, held the generally accepted views as to the dangerous effects of anæsthetics, he must have somewhat changed them. The second commission was composed of Dr. Laurie as president, and Drs. Brunton, Bomford, and Rustomji as members. The members of the first commission also aided in the work. After numerous experiments the practical conclusions of the committee are as follows. When administering anæsthetics the recumbent posture of the patient on the back and absolute freedom of respiration are essential. If from any cause this position cannot be maintained, the utmost attention to respiration is necessary. An apparatus ought not to be used, as, being made to fit the face, it must tend to produce a certain amount of asphyxia. Moreover, it takes attention required elsewhere. At the commencement of inhalation care should be taken, by not holding the cap or cone too close over the mouth and nose, to avoid exciting struggling or holding of the breath. When quiet breathing is ensured all that is necessary is to watch the cornea, and to see that respiration is not interfered with. Struggling is best prevented in adults by making them blow hard after each inspiration. In any struggling person, especially in children, it is essential to remove the inhaler after the first or second deep inspiration. The patient is, as a rule, ready for the operation to be commenced when unconscious winking is not produced by touching the cornea. The anæsthetic should never be pushed till respiration stops, but when the cornea is insensitive the patient should be kept gently under by occasional inhalations, and not allowed to recover and renew the stage of struggling and resistance. The administrator should be guided as to the effect entirely by the respiration. His only object while producing anæsthesia is to see the respiration is not interfered with. If practicable, both chest and abdomen should be exposed, so that the respirative movement can be seen by the administrator. If anything interferes with the respiration, however slightly, even if this occurs at the very commencement, if breath is held, or if there is sterton, the inhalation should be stopped until the breathing is natural. If breathing becomes embarrassed, the lower jaw should be pulled or pushed from behind the angles, so that the lower teeth project in front of the upper, which raises the epiglottis and frees the larynx. If by any accident respiration stops, artificial respiration should be commenced, while an assistant lowers the head and draws forward the tongue with forceps. A small dose of morphia may be injected sub-cutaneously before chloroform inhalation, as it helps to keep the patient in a state of anæsthesia. The above conclusions do not differ materially from those of Dr. Laurie and the members of the first commission. It seems now conclusively proved that the danger of the administration of anæsthetics does not consist in an action on the heart, at least, primarily, but on a stoppage of respiration. On this point a contemporary observes that "the facts brought forward by the first commission seemed to us insufficient to overcome the conviction of many observers both in this country and abroad, whose researches seemed to prove that one of the dangers of chloroform was paralysis of the heart." The profession is generally slow to recognise the work of Anglo-Indian surgeons, but in this matter the conclusions arrived at have been the result of a long series of experimental researches, conducted by two commissions, an eminent London physiologist being a member of the latter. We must congratulate Dr. Laurie on having established his position so thoroughly, and we cannot but feel grateful to His Highness the Nizam, whose liberality enabled the commission to so thoroughly investigate this important subject.

